# Global Scientific Trends in Virtual Reality for Pain Treatment From 2000 to 2022: Bibliometric Analysis

**DOI:** 10.2196/48354

**Published:** 2023-11-14

**Authors:** Xun Yang, Sen Zhong, Sheng Yang, Meng He, Xu Xu, Shisheng He, Guoxin Fan, Lijun Liu

**Affiliations:** ^1^Department of Traumatic Orthopedics, Shenzhen Second People’s Hospital (The First Affiliated Hospital, Shenzhen University; Shenzhen Translational Medicine Institute), Shenzhen, China; 2 Department of Orthopedic, School of Medicine, Spinal Pain Research Institute, Shanghai Tenth People’s Hospital, Tongji University, Shanghai, China; 3 Department of Pain Medicine, National Key Clinical Pain Medicine of China, Huazhong University of Science and Technology Union Shenzhen Hospital, Shenzhen, China

**Keywords:** virtual reality, pain management, bibliometrics, research trends, CiteSpace, VOSviewer

## Abstract

**Background:**

Virtual reality (VR) is a computer simulation technique that has been increasingly applied in pain management over the past 2 decades.

**Objective:**

In this study, we used bibliometrics to explore the literature on VR and pain control, with the aim of identifying research progress and predicting future research hot spots.

**Methods:**

We extracted literature on VR and pain control published between 2000 and 2022 from the Web of Science Core Collections and conducted bibliometric analyses. We analyzed the publication and citation trends in the past 2 decades, as well as publication and citation analyses of different countries, institutions, journals, and authors. For references, we conducted cocitation and burst analyses. For keywords, we conducted co-occurrence, clustering, timeline view, and citation burst analyses.

**Results:**

Based on 1176 publications, we found that there was a continuous increase in publication and citation volumes, especially in the last 5 years. The United States was the most representative country, and the University of Washington was the most representative institution, with both having the most publications and citations. The most popular journal in this field was *Burns*, and Hoffman HG was the most productive author, leading many studies on patients with burn pain. The reference with the most citation burst was a study on the verification of new hardware in pain control. The keywords with the highest citation bursts related to various situations of pain such as “burn pain,” “wound care,” “low back pain,” and “phantom limb.”

**Conclusions:**

VR has been applied in various clinical situations for pain management, among which burns and pediatric surgery have achieved satisfactory results. We infer that VR will be extended to more clinical pain situations in the future, such as pain control in wound care, low back pain, and phantom limb pain. New research hot spots will include the development of software and hardware to improve the immersive experience of VR for pain control. However, our work was based solely on English literature from the Web of Science database. For future studies, we recommend that researchers explore literature from multiple databases to enhance the scope of their research.

## Introduction

Pain is an unpleasant sensory and emotional experience that can affect patients’ quality of life. Any form of harmful irritation has the potential to cause pain, and various medical procedures, including venipuncture and wound care, can result in temporary discomfort for patients. Moreover, chronic pain can have an even more substantial impact on individuals. It has been reported to affect more than 30% of people worldwide, and epidemiological studies have estimated that the prevalence of chronic pain is between 18% and 34.5% [1-3]. To a certain extent, continuous pain can adversely affect the patients’ family relationships and become the cause of both heavy social burden and economic burden [4-7]. The main clinical treatment for pain is medication, including paracetamol, opioids, etc. In addition, there are other interventions to relieve pain such as psychological interventions, acupuncture, massage, and spinal cord stimulation [8]. Because of the strong influence of psychological factors on pain perception, psychological intervention has become an important means of adjuvant drug treatment [9].

Virtual reality (VR) is an emerging technology in the psychological intervention for pain, which immerses users in a virtual 3D environment through a wearable screen [10]. In recent years, research has demonstrated that VR can effectively control pain by shifting attention [11]. Some researchers believe that VR influences memory, emotion, and attention, which can help alleviate pain [12]. Functional magnetic resonance imaging has provided evidence that VR can effectively reduce the activity of brain regions associated with pain [13], similar to the effects of opioids [14]. Due to its action mechanism, VR has shown significant efficacy in controlling acute pain and has been used in the management of pain during procedures such as burn wound care and acupuncture [15,16]. Additionally, VR has been applied to chronic pain management, where it creates a sense of presence in a new environment, improves mood, and diverts attention, ultimately reducing pain levels [17]. Chronic conditions such as chronic low back pain [18], chronic cervical pain [19], fibromyalgia [20], complex regional pain syndrome [21], and chronic neuropathic pain have also been targeted with VR-based interventions [22,23].

Despite the growing scientific literature on the use of VR in pain management, there is still a lack of studies on its development trends and hot spots, which can provide scientific guidance for researchers in this area. Bibliometrics is an analytical method that can generate a comprehensive view or quantitative parametric analysis of an entire research field or a specific scientific application area [24-26]. This study aimed to analyze the application of VR in the field of pain management in the past 2 decades using bibliometrics, to provide researchers with an overall research development trend and to help guide future research directions.

## Methods

### Search Strategy

We searched publications from the Web of Science Core Collection (WoSCC) for analysis. The search strategy was “((TS= (virtual reality)) AND ((TS=(pain)) OR TS= (painful))).” The search was conducted in April 2022.

### Included and Excluded Criteria

**Figure 1.**
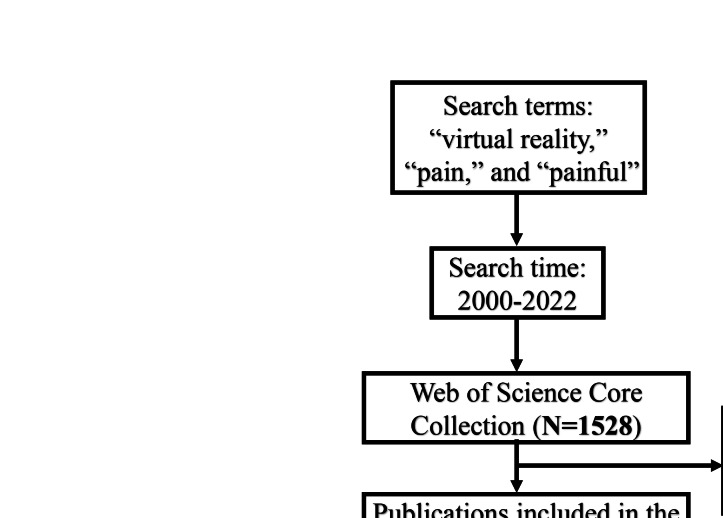
The screening process of the selected literature in this study.

This study included articles and reviews that were published from 2000 to 2022. The following were excluded: (1) non-English literature and (2) publications that are books, book chapters, meeting abstracts, letters, conference proceedings, editorial materials, etc. The screening process is shown in Figure 1.

### Data Export and Analysis

We obtained the information of these publications from the WoSCC through the above search strategy. CiteSpace (version 5.8.R3; Drexel University) and VOSviewer (version 1.6.11.0; Centre for Science and Technology Studies, Leiden University) were used for further data analysis. Quantitative and qualitative analyses were performed and presented in this study, including the following:

1. Annual trends of the publications and citations: these present the general trends in this field.
2. Publication and citation analyses of different countries and institutions: these aim to find the countries or institutions that have contributed more to this field and their partnerships. Researchers may choose to learn from or collaborate with them.
3. Publication and cocitation analyses of different journals: if a study published in journal A and another study published in journal B are both cited by a study published in journal C, then journal A and journal B are considered to have a cocitation relationship and the cocitation count is increased by 1. The definition also extends to the cocitation analysis of authors and references, which is described in further detail below. Journals with a higher number of publications and cocitations are considered as more important journals in the field. Researchers can pay attention to these journals to identify the research progress or publish their study in these journals in the future.
4. Publication and cocitation analyses of different authors: by identifying authors with a high volume of publications or cocitations, researchers can potentially connect with leading experts and stay informed about their research directions and progress.
5. Cocitation and citation burst analyses of references: references with a high number of cocitations can provide insights into the research hot spots of a field. These references are widely recognized and cited by authors in the field, indicating their relevance and significance within the research community. Researchers can use these references to identify key topics and focuses within the field. The citation burst analysis is an indication of the increase regarding researchers’ attention to a reference over a given period of time, which is a very important indicator for identifying research hot spot trends.
6. Keywords analysis (co-occurrence, clustering, timeline view, and citation burst): we presented the keywords in the field based on co-occurrence and obtained the research hot spots in the field through the keywords that appear more frequently. Subsequently, those keywords were divided into several clusters, with similar keywords in each cluster, to capture the main research topics. By tracking these keywords through a timeline perspective, we gained a better understanding of the development and evolution in this field over time. Finally, by the analysis of citation bursts, we identified the research hot spots and trends in the field.

## Results

### Publication and Citation Trends

**Figure 2.**
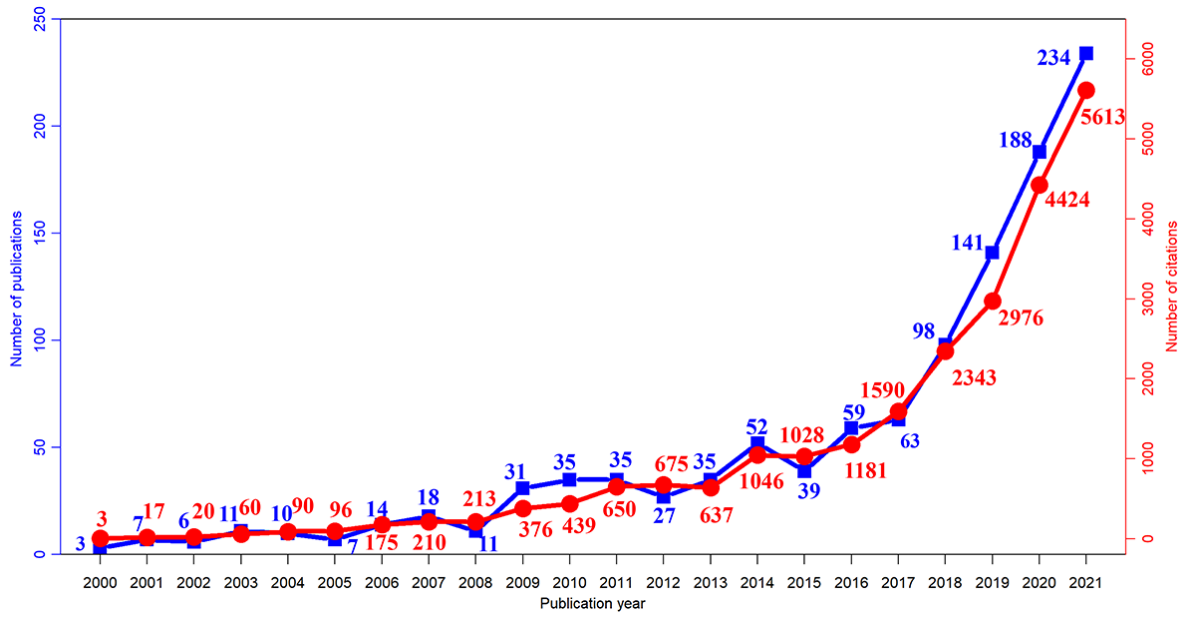
Annual trends of publications and citations.

A total of 1528 publications were retrieved from the WoSCC. In all, 1176 publications were used for further analysis after excluding 41 non-English publications and 311 other publication types. Time trend analysis showed that from 2000 to 2021, the number of published papers increased from 3 to 234, which was statistically significant (2-tailed *t*_22_=4.061, *P*=.001; Figure 2). The annual citation frequency had also increased from 3 times in 2000 to 5613 times in 2021, which indicated a similarly significant growth (2-tailed *t*_22_=3.565, *P*=.002). In addition, publications and citations in the past 5 years showed a particularly rapidly growth, with an average annual increase of 42.75 papers and 1005.75 citations.

### Distribution of Countries and Institutions

**Figure 3.**
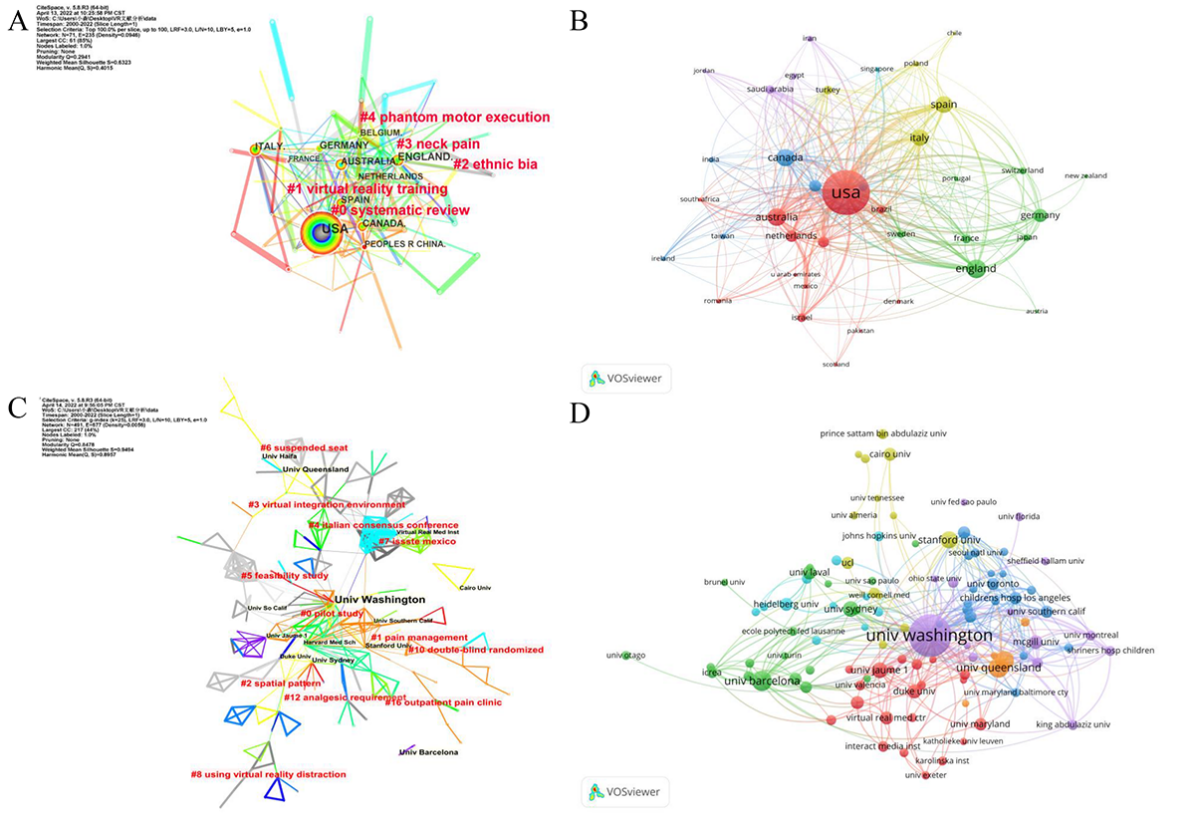
Distribution of countries and institutions. (A) Cooperation mapping of different countries. (B) Citation mapping of different countries. (C) Cooperation mapping of different institutions. (D) Citation mapping of different institutions.

In this study, all the included publications came from 69 countries. The top 11 countries with the most publications are showed in Figure 3A (each node in the figure represents a country). Of these countries, the United States (n=382) had the most publications, followed by England (n=97), Australia (n=89), Canada (n=86), and Spain (n=81). Furthermore, the country citation analysis is shown in Figure 3B. The lines between countries represent the mutual citation relationship between them (Figure 3B); more frequent citations are represented by thicker lines. A total of 1674 institutions participated in this research area, as shown in Figure 3C. In this figure, the institution that generated the most publications was the University of Washington (n=70), followed by the University of Barcelona (n=19), University of Queensland (n=18), University of Haifa (n=14), and University of Sydney (n=14). Similarly, we also analyzed the citation of institutions (Figure 3D). The top 5 countries and institutions with the most citations are summarized in Table S1 in Multimedia Appendix 1. Moreover, clustering analyses of countries and institutions are summarized in Tables S2 and S3 in Multimedia Appendix 1. A total of 5 and 12 clusters were found for countries and institutions, respectively. The top 5 countries and institutions in each cluster are shown in Tables S2 and S3 in Multimedia Appendix 1.

### Distribution of Journals

**Figure 4.**
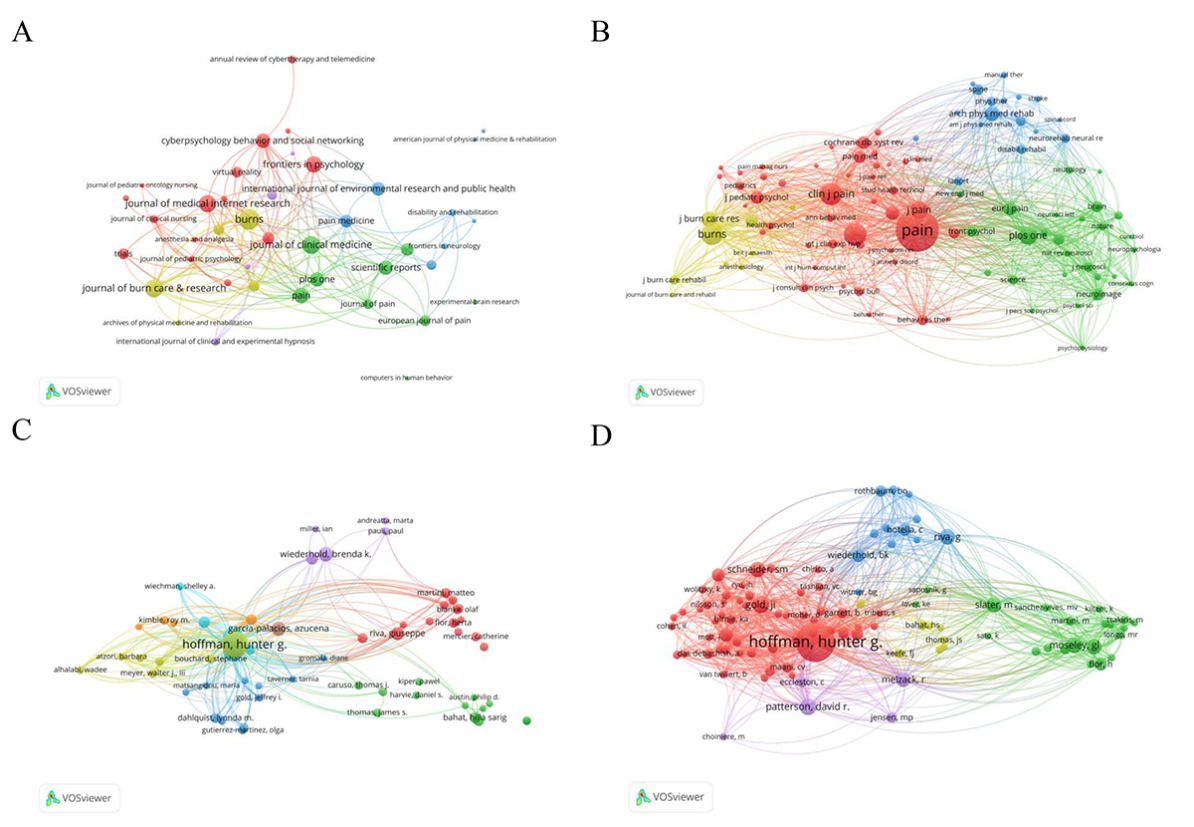
Distribution of journals and authors. (A) Publication mapping of different journals. (B) Cocitation mapping of different journals. (C) Cooperation mapping of different authors. (D) Cocitation mapping of different authors.

The included 1176 publications on pain and VR research came from 720 academic journals, of which the top 10 journals with the most publications accounted for 15.31% (180/1176) of the total publications, which can be considered as authoritative journals or mainstream journals in this field. Figure 4A shows the visualization results of the publications by different journals. Each node represents a journal, and a larger node indicates greater volume of publications. The cocitation relationship between journals is shown in Figure 4B. The line connecting the nodes indicates that there was a cocitation relationship between the 2 nodes. The top 10 journals with the most publications and cocitations are summarized in Table 1. Among the top 10 journals, 60% (n=6) were from the Q1 quartile and 30% (n=3) were from the Q2 quartile.

**Table 1.** The top 10 journals with the most publications and cocitations.

Ranking		Journal	JCR^a^ category	Category rank in 2020	Category quartile in 2020	IF^b^ in 2020	Publications or cocitations, n
**Publications**							
	1	*Burns*	Critical care medicine (SCIE^c^) Dermatology (SCIE) Surgery (SCIE)	23/36 37/69 88/211	Q3 Q3 Q2	2.744	23
	2	*Journal of Burn Care and Research*	Critical care medicine (SCIE) Dermatology (SCIE) Surgery (SCIE)	30/36 52/69 147/211	Q4 Q4 Q3	1.845	20
	3	*Journal of Clinical Medicine*	Medicine, general & internal (SCIE)	39/167	Q1	4.242	20
	4	*Journal of Medical Internet Research*	Health care sciences & services (SCIE) Medical informatics (SCIE)	10/107 5/30	Q1 Q1	5.428	19
	5	*Frontiers in Psychology*	Psychology, multidisciplinary (SSCI^d^)	43/140	Q2	2.988	18
	6	*Pain*	Anesthesiology (SCIE) Clinical neurology (SCIE) Neurosciences (SCIE)	4/33 23/208 39/273	Q1 Q1 Q1	6.961	17
	7	*Cyberpsychology Behavior and Social Networking*	Psychology, social (SSCI)	14/65	Q1	4.157	16
	8	*PLoS One*	Multidisciplinary sciences (SCIE)	26/72	Q2	3.24	16
	9	*Scientific Reports*	Multidisciplinary sciences (SCIE)	17/72	Q1	4.38	16
	10	*International Journal of Environment Research and Public Health*	Environmental sciences (SCIE) Public, environmental & occupational health (SCIE) Public, environmental & occupational health (SSCI)	118/274 68/203 42/176	Q2 Q2 Q1	3.39	15
**Cocitations**							
	1	*Pain*	Anesthesiology (SCIE) Clinical neurology (SCIE) Neurosciences (SCIE)	4/33 23/208 39/273	Q1 Q1 Q1	6.961	2341
	2	*Cyberpsychology & Behavior*	Communication (SSCI) Psychology, applied (SSCI)	1/72 11/73	Q1 Q1	2.71	946
	3	*Clinical Journal of Pain*	Anesthesiology (SCIE) Clinical neurology (SCIE)	16/33 99/208	Q2 Q2	3.442	930
	*4*	*Burns*	Critical care medicine (SCIE) Dermatology (SCIE) Surgery (SCIE)	23/36 37/69 88/211	Q3 Q3 Q2	2.744	914
	5	*PLoS One*	Multidisciplinary sciences (SCIE)	26/72	Q2	3.24	728
	6	*Journal of Pain*	Clinical neurology (SCIE) Neurosciences (SCIE)	34/208 57/273	Q1 Q1	5.828	688
	7	*Journal of Burn Care ＆ Research*	Critical care medicine (SCIE) Dermatology (SCIE) Surgery (SCIE)	30/36 52/69 147/211	Q4 Q4 Q3	1.845	558
	8	*Archives of Physical Medicine and Rehabilitation*	Rehabilitation (SCIE) Sport sciences (SCIE)	5/68 23/88	Q1 Q2	3.966	528
	9	*Cyberpsychology Behavior and Social Networking*	Psychology, social (SSCI)	14/65	Q1	4.157	472
	10	*European Journal of Pain*	Anesthesiology (SCIE) Clinical neurology (SCIE) Neurosciences (SCIE)	13/33 75/208 121/273	Q2 Q2 Q2	3.934	467

a JCR: Journal Citation Reports.

b IF: impact factor.

c SCIE: Science Citation Index Expanded.

d SCCI: Social Sciences Citation Index.

### Distribution of Authors

The relationships between author and citation are showed in Figure 4C. Each node represents an author and the size of the node indicates the publication counts of the author. The lines between the nodes represent the citation relationship between authors: the thicker the line, the more citations there are between the 2 authors. Table 2 showed the top 10 authors with the most publications and their cocitations. For better visualization, we only included authors with more than 30 cocitations, and a total of 153 authors were included (Figure 4D). The top 10 authors with the most publications came from 4 countries (the United States, Spain, Italy, and Israel), and 7 (70%) out of 10 are American authors.

**Table 2.** The top 10 authors with the most publications and cocitations.

Ranking		Author	Publications or cocitations, n	Country
**Publications**				
	1	Hoffman HG	47	United States
	2	Patterson DR	32	United States
	3	Sharar SR	28	United States
	4	Wiederhold BK	17	United States
	5	Garcia-Palacios A	16	Spain
	6	Riva G	14	Italy
	7	Wiederhold MD	13	United States
	8	Jensen MP	11	United States
	9	Bahat HS	11	Israel
	10	Carrougher GJ	10	United States
**Cocitations**				
	1	Hoffman HG	1561	United States
	2	Gold JI	275	United States
	3	Patterson DR	270	United States
	4	Moseley GL	253	Australia
	5	Riva G	236	Italy
	6	Ramachandran VS	227	India
	7	Schneider SM	225	United States
	8	Slater M	222	Spain
	9	Melzack R	208	Canada
	10	Dahlquist L	168	United States

### Distribution of References

**Figure 5.**
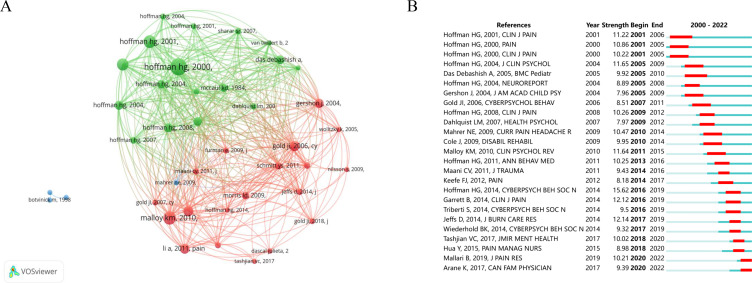
Distribution of references. (A) Cocitation mapping of different references. (B) Top 25 references with the strongest citation bursts.

We analyzed a total of 36,224 references from 1176 papers. The most frequently cited and cocited papers are summarized in Table 3. The cocitation relationships are visualized in Figure 5A. It is worth noting that the top 10 cocited references were all from the United States, and 6 of them were from Hoffman HG. We found that all the papers that were cocited the most were about pain control using VR technology.

**Table 3.** The top 10 papers with the most citations and cocitations.

Ranking		Citations or cocitations, n	Title	Authors and year	Journal	Country
**Citations**						
	1	435	Virtual Reality in Neuroscience Research and Therapy	Bohil et al [27], 2011	*Nature Reviews Neuroscience*	United States
	2	304	Don’t Fear ‘Fear Conditioning’: Methodological Considerations for the Design and Analysis of Studies on Human Fear Acquisition, Extinction, and Return of Fear	Lonsdorf et al [28], 2017	*Neuroscience and Biobehavioral Reviews*	Germany
	3	274	Virtual Reality as an Adjunctive Pain Control During Burn Wound Care in Adolescent Patients	Hoffman et al [29], 2000	*Pain*	United States
	4	254	Biofeedback in Rehabilitation	Giggins et al [30], 2013	*Journal of Neuroengineering and Rehabilitation*	Ireland
	5	253	The Effectiveness of Virtual Reality Distraction for Pain Reduction: A Systematic Review	Malloy and Milling [31], 2010	*Clinical Psychology Review*	United States
	6	242	Keeping Pain in Mind: A Motivational Account of Attention to Pain	van Damme et al [32], 2010	*Neuroscience and Biobehavioral Reviews*	Belgium
	7	239	Transcontinental Robot-Assisted Remote Telesurgery: Feasibility and Potential Applications	Marescaux et al [33], 2002	*Annals of Surgery*	France
	8	220	Use of Virtual Reality for Adjunctive Treatment of Adult Burn Pain During Physical Therapy: A Controlled Study	Hoffman et al [9], 2000	*Clinical Journal of Pain*	United States
	9	200	Effectiveness of Virtual Reality-Based Pain Control With Multiple Treatments	Hoffman et al [34], 2001	*Clinical Journal of Pain*	United States
	10	199	Virtual Reality as an Adjunctive Non-Pharmacologic Analgesic for Acute Burn Pain During Medical Procedures	Hoffman et al [35], 2011	*Annals of Behavioral Medicine*	United States
**Cocitations**						
	1	192	Virtual Reality as an Adjunctive Pain Control During Burn Wound Care in Adolescent Patients	Hoffman et al [29], 2000	*Pain*	United States
	2	149	The Effectiveness of Virtual Reality Distraction for Pain Reduction: A Systematic Review	Malloy and Milling [31], 2010	*Clinical Psychology Review*	United States
	3	142	Effectiveness of Virtual Reality-Based Pain Control With Multiple Treatments	Hoffman et al [34], 2001	*Clinical Journal of Pain*	United States
	4	135	Use of Virtual Reality for Adjunctive Treatment of Adult Burn Pain During Physical Therapy: A Controlled Study	Hoffman et al [9], 2000	*Clinical Journal of Pain*	United States
	5	116	Virtual Reality as an Adjunctive Non-Pharmacologic Analgesic for Acute Burn Pain During Medical Procedures	Hoffman et al [35], 2011	*Annals of Behavioral Medicine*	United States
	6	114	Effectiveness of Virtual Reality for Pediatric Pain Distraction During IV Placement	Gold et al [36], 2006	*Cyberpsychology & Behavior*	United States
	7	112	Virtual Reality and Pain Management: Current Trends and Future Directions	Li et al [37], 2011	*Pain Management*	United States
	8	104	A Pilot and Feasibility Study of Virtual Reality as a Distraction for Children With Cancer	Gershon et al [38], 2004	*Journal of the American Academy of Child and Adolescent Psychiatry*	United States
	9	102	Modulation of Thermal Pain-Related Brain Activity With Virtual Reality: Evidence From fMRI	Hoffman et al [13], 2004	*Neuroreport*	United States
	10	102	Virtual Reality Pain Control During Burn Wound Debridement in the Hydrotank	Hoffman et al [39], 2008	*Clinical Journal of Pain*	United States

Figure 5B [9,11,13,29,31,34-36,38-54] shows the top 25 strongest citation bursted between 2000 and 200enting more citations and green ones representing fewer citations. A burst of reference means that this paper has been cited more frequently by other papers during this period. From the burst analysis of the references, we found that new directions and new hot spots in this field were constantly emerging and research interest in VR and pain had grown substantially. The burst of reference citations first started from 3 papers written by Hoffman HG in 2001, of which 2 were published in the *Clinical Journal of Pain* and the other in *Pain*. After a lapse of 14 years, the article published by Hoffman HG in *Cyberpsychology Behavior and Social Networking* in 2014 became the most persuasive one. These 3 journals were also among the top 10 journals in the field of VR and pain. Moreover, 8 of the top 25 strongest citation reference were published by Hoffman HG, which was sufficient to justify that Hoffman HG was a pioneer and leader in this field. The latest emerging authors were Mallari B and Arane K, who had published articles in the *Journal of Pain Research* and *Canadian Family Physician*, and these articles had received high citations recently.

### Keyword Analysis

**Figure 6.**
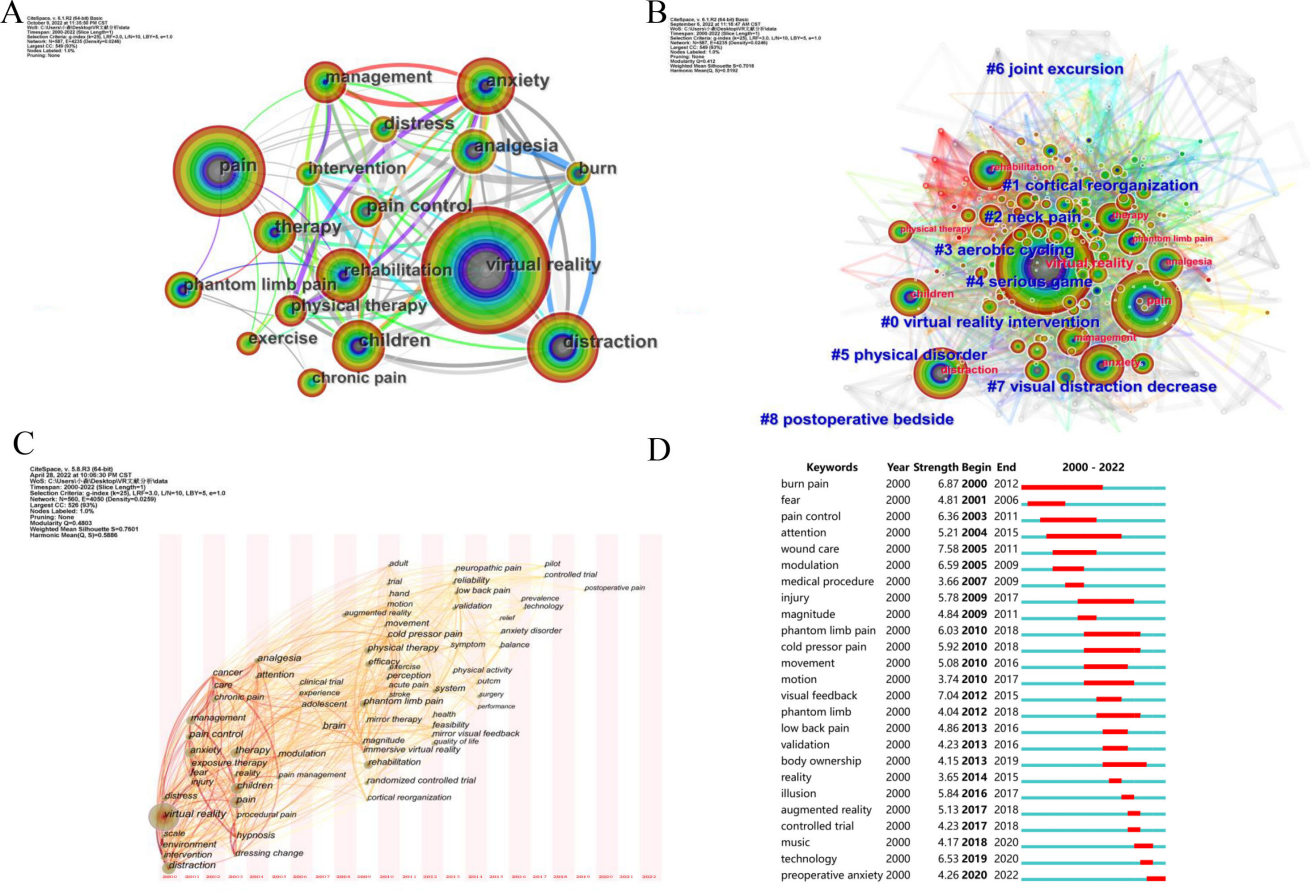
Keyword analyses. (A) Co-occurrence mapping of different keywords. (B) Clustering mapping of different keywords. (C) Timeline view mapping of different keywords. (D) Top 25 keywords with the strongest citation bursts.

The keyword co-occurrence analysis is shown in Figure 6A. The top 10 keywords and their centrality are summarized in Table S4 in Multimedia Appendix 1. The keyword with the highest number of occurrences was “virtual reality” with the highest centrality of 0.09, followed by “pain” with 348 occurrences and a centrality of 0.07.

A total of 9 clusters were identified in the clustering analysis, and they are shown in Figure 6B. The *Q* value is 0.412>0.3, and the *S* value is 0.7018>0.7, indicating that the clusters were credible. The top 10 keywords in each cluster are shown in Table S5 in Multimedia Appendix 1. In the cluster, labels were derived from the keywords; a smaller cluster number indicates that the cluster contains more keywords.

The timeline view analysis is shown in Figure 6C. For better visualization, we only showed keywords with more than 18 occurrences. Each node represents a keyword, and only the year that the keyword first appeared was counted. The size of the nodes indicates the frequency of the occurrence. The lines between nodes refer to 2 keywords appearing in the same paper or several papers. For examples, “virtual reality” first appeared in 2000, and the node is the largest, indicating that “virtual reality” had the most occurrences from 2000 to 2022. “Pain” first appeared in 2003, and there is a connection between “virtual reality” and “pain,” indicating that these 2 keywords appeared in 1 or several papers in 2003 at the same time.

The citation burst analysis of the keywords is shown in Figure 6D. The most cited keyword was “wound care,” followed by “visual feedback,” “burn pain,” and “modulation,” indicating that the research on pain relief using VR technology was very popular in the field of burn pain. The latest hot keyword was “preoperative anxiety.”

## Discussion

### Overview

With high incidence and prevalence, chronic pain has become the cause of both heavy social burden and economic burden. VR is a computer simulation technique that has been shown to help patients manage their pain, reducing unnecessary medication use and the numerous side effects. In this study, we analyzed the development process of VR in the field of pain management, as well as research hot spots and future trends. Our results showed that publications in this field grew rapidly, and the United States is now leading in this field. According to our research, the hot spot in this field was burn-related pain, and the representative keywords were “virtual reality,” “burn,” “surgery” and “pain control.” This study provides systematic guidance and potential research directions for researchers in this area.

### Principal Findings

VR was first popularized for pain control by Hoffman HG in 2000 [29,55]. Subsequently, publications in this field increased slowly from 2000 to 2017. After 2017, the publications increased rapidly from 63 in 2017 to 234 in 2021, with an average annual increase of 42.75 papers. We infer that there may be 2 reasons for this. First, according to the hype cycle of emerging technologies produced by the Gartner Group in 2015 [56], VR just ended the trough of disillusionment period in 2015 and was in the slope of enlightenment period. At that time, more and more organizations carried out in-depth research on VR technology [57]. The second reason is that as many technology companies such as Facebook, Sony, and other companies made huge investments in the VR field around 2016, VR technology has continued to improve. Additionally, there have been more ways for researchers to obtain VR technology while the cost has also become much lower [58]. In the future, with the continuous innovation of VR technology, we believe that the research on VR technology in medical treatment, including pain control, will become more and more popular. In terms of the global publications, the United States had published a total of 382 articles, accounting for about 32.5% of the 1176 selected articles, which indicates that the United States is the absolute leader in this field. In terms of the number of citations, US researchers topped the list with 12,454 citations, indicating that literature published from the United States are authoritatively reliable in the field of VR pain management, which is inseparable from the leading level of VR technology research. Other countries still have great potential for research in this field on the basis of this citation analysis.

The quality of articles published in this field was also high. Overall, 60% of the top 10 journals were from the Q1 quartile. The top 5 journals with the highest number of publications were *Burns*, *Journal of Burn Care & Research*, *Journal of Clinical Medicine*, *Journal of Medical Internet Research*, and *Frontiers in Psychology*. To sum up, papers in these journals were mostly about burn pain, which is the first application area of VR in pain control [9,29]. Hoffman et al [59,60] have done a lot on the application of VR in burns, and they have confirmed that VR is effective and feasible in pain control of patients with burn pain. However, the mechanism of VR analgesia has not yet been fully elucidated. Whether the analgesic effect can be improved through interaction in the field of burns is a research trend, and there is still a lot of research space in the field of burns [60].

### Research Hot Spots

Combined with literature cocitation analysis and keyword co-occurrence analysis, the current research focus in the field revolves around 2 main areas: the control of burn-related pain and pain control during medical operations in children. Two important studies conducted by Hoffman et al [9,29] in 2000 demonstrated that VR can reduce pain perception in patients with burn pain during physical therapy and alleviate pain during burn wound care. Subsequent clinical experiments have consistently confirmed the pain-reducing effects of VR in procedures such as excision and hydrotherapy debridement for patients with burn pain [39,61-63]. Furthermore, Hoffman et al [64] provided additional evidence for VR’s pain control capabilities in patients with burn pain by using functional magnetic resonance imaging to demonstrate a reduction in pain-related brain activity while using VR devices. Lee et al [65] used functional near infrared spectroscopy to analyze cerebral blood flow in patients with burn pain during VR experiences. They discovered a significant increase in oxygenated hemoglobin in the prefrontal lobe, suggesting that VR activates the prefrontal lobe to inhibit pain sensations.

Researchers have also been interested in understanding the factors that influence VR’s pain control in patients with burn pain. Sharar et al [66] found that VR’s analgesic effect remains consistent regardless of factors such as age, gender, race, burn wound size, or duration of treatment. However, a comparison study by Hoffman et al [67] showed that VR headsets with wider viewing angles result in a more immersive experience and better pain control compared to headsets with narrower viewing angles. In summary, the pain-relieving benefits of VR for patients with burn pain have been consistently validated, and future efforts should focus on optimizing the VR experience to further enhance pain control efficacy.

Moreover, VR has shown promising results in pain relief during medical procedures for pediatric patients. Beyond its application in burn-related scenarios, VR has been used for venipuncture, intravenous catheterization [68], lumbar punctures [69], and even preoperative procedures [70] to alleviate anxiety and minimize procedure-related pain in children. Compared to adults, VR is particularly effective in capturing the attention of children and can be applied in various clinical scenarios. Recent research has explored the use of VR for preoperative anxiety relief, postoperative pain management [71], pain control during vaccinations [72], anesthesia and tooth extraction [73], as well as pain management during emergency sutures [73,74]. In the future, VR is poised to play an important role in various pediatric medical operations.

### Research Trends and Directions

The role of VR in pain control has been proven, and future research in this field aims to promote the application scenarios of VR pain control and enhance the user experience for patients to improve the analgesic effect. At present, in addition to the application of treating patients with acute burn pain, the analgesic effect of VR has been applied in the first stage of labor analgesia [75], wound care [76], cystoscopy [77], oral surgery [78], and postoperative pain management [79]. However, further studies are needed to confirm its effectiveness in different clinical scenarios such as gastroenteroscopy and puncture surgery. Patients with chronic pain, including chronic cancer pain, chronic neuropathic pain, and chronic low back pain, can also benefit from VR devices, which can provide pain relief and improve mood [80-83]. To manage chronic pain effectively, there is a need to develop more diverse VR software that caters to the needs of patients.

Improving the patient experience will also be a key focus for future research. As an immersive device, VR presents certain challenges. First, hardware equipment needs to be enhanced to provide a more interactive VR experience, as suggested by Yeo et al [84]. Second, the development of high-quality VR software specific to different pain types is crucial for enhancing the patient experience. Additionally, creating appropriate VR scenarios and games tailored to patients’ needs will contribute to a better patient experience.

In conclusion, this study conducted a 2-decade bibliometric analysis of research on VR interventions in pain management. Currently, the research of VR in the field of pain has received extensive attention, and satisfactory results have been achieved in a series of fields such as burn pain and pediatrics medical procedure–related pain. It is remarkable that burn pain control is still a research hot spot in this field. The emerging trends include the application of VR in other departments related to pain and improving VR technology to enhance the patient experience. However, the mechanism of pain control with VR is not yet understood, and researchers are constantly working in this field, which will guide the development of more effective VR pain management technology.

### Strengths and Limitations

This is the first bibliometric analysis of VR research in the field of pain. We provided scientific information in this field and predicted research hot spots and research trends to summarize the research status in this field and further promote the development of this field.

However, there are multiple limitations in our research. First, we only analyzed publications in the WoSCC and failed to exhaust all the publications on VR and pain control. Second, due to limitations of the analysis tools, we only analyzed the papers published in English and discarded other valuable books as well as non–article and review literature such as conference papers. Last but not least, our retrieval methods also have shortcomings, but they simplified the literature so that it is more in line with our theme, and simplicity was conducive to the development of our analysis and research. However, we still hope that future researchers can use more search terms to include some fringe literature in the analysis scope, which may be a useful recommendation for future treatment in this field.

### Conclusions

Through bibliometric analysis and visualization, we provided an overview analysis of the field of VR for pain control. There were more than 60 countries participating in this field, and the relative research had been growing rapidly in recent years. The United States and the University of Washington were the representative country and institution, and Hoffman HG was the pioneer in this field. Through the analysis, we identified the hot spots in this research field, which included burn pain and pediatric surgery pain. More attention will be paid to the application of VR in various pain situations in the future, and more diversified software and hardware will be developed to improve the immersive experience of patients, making VR more distracting and more effective at reducing pain.

## Supplementary material

10.2196/48354 Multimedia Appendix 1 The top 5 countries and institutions with the most citations, the top 5 countries in each country cluster, the top 5 institutions in each institution cluster, the top 10 co-occurrence keywords and the centrality, and the top 10 keywords in each keyword cluster.

## Abbreviations

VR: virtual reality
WoSCC: Web of Science Core Collection
