# Supplementary material for: Global Scientific Trends in Virtual Reality for Pain Treatment From 2000 to 2022: Bibliometric Analysis
Source: JMIR Serious Games. 2023 Nov 14;11:e48354. doi: 10.2196/48354 (PMC10686536; doi:10.2196/48354)
Supplement: Multimedia Appendix 1 [file games-v11-e48354-s001.docx]

**Multimedia Appendix 1**

Table S1. The top five countries and institutions with the most citations.

Table S2. The top five countries in each country cluster.

Table S3. The top five institutions in each institution cluster.

Table S4. The top ten co-occurrence keywords and the centrality.

Table S5. The top ten keywords in each keyword cluster.

Table S1. The top five countries and institutions with the most citations.

| Ranking | Country | Frequency | Insitution | Abbreviations | Frequency |
| --- | --- | --- | --- | --- | --- |
| 1 | USA | 12454 | Washington State University | Univ Washington | 4339 |
| 2 | Australia | 2064 | Duke University | Duke Univ | 888 |
| 3 | England | 1952 | The University of Queensland | Univ Queensland | 743 |
| 4 | Canada | 1933 | University of Southern California | Univ So Calif | 709 |
| 5 | Italy | 1908 | Jaume I University | Univ Jaume I | 589 |

Table S2. The top five countries in each country cluster.

| Cluster label | Country | Publications |
| --- | --- | --- |
| #0 systematic review | USA | 382 |
|  | Germany | 58 |
|  | Peoples R China | 47 |
|  | South korea | 28 |
|  | Switzerland | 22 |
| #1 virtual reality training | Spain | 81 |
|  | Italy | 79 |
|  | Saudi arabia | 26 |
|  | Egypt | 13 |
|  | India | 13 |
| #2 ethnic bia | England | 97 |
|  | Scotland | 8 |
|  | Finland | 4 |
|  | Cyprus | 3 |
|  | North ireland | 3 |
| #3 neck pain | Australia | 89 |
|  | France | 31 |
|  | Israel | 30 |
|  | Brazil | 25 |
|  | China-Taiwan | 15 |
| #4 phantom motor execution | Canada | 86 |
|  | Netherlands | 45 |
|  | Belgium | 38 |
|  | Sweden | 24 |
|  | Ireland | 12 |

Table S3. The top five institutions in each institution cluster.

| Cluster label | Institution | Publications | Cluster label | Institution | Publications |
| --- | --- | --- | --- | --- | --- |
| #0 pilot study | Univ Washington | 70 | #6 suspended seat | Univ Queensland | 18 |
|  | Univ Jaume I | 10 |  | Univ Haifa | 14 |
|  | Univ Texas Med Branch | 7 |  | La Trobe Univ | 4 |
|  | King Abdulaziz Univ | 6 |  | Monash Univ | 4 |
|  | Univ Ottawa | 6 |  | Griffith Univ | 4 |
| #1 pain management | Stanford Univ | 14 | #7 issste Mexico | Virtual Real Med Ctr | 8 |
|  | Univ Southern Calif | 9 |  | Interact Media Inst | 8 |
|  | Harvard Med Sch | 9 |  | Hosp Angeles Mexico | 2 |
|  | Childrens Hosp Los Angeles | 8 |  | Univ Panamer Mexico City | 2 |
|  | Univ Maryland | 8 |  | ISSSTE | 2 |
| #2 spatial pattern | Univ Sydney | 13 | #8 using virtual reality distraction | Univ Birmingham | 6 |
|  | Duke Univ | 10 |  | Imperial Coll London | 6 |
|  | Heidelberg Univ | 7 |  | Univ Exeter | 4 |
|  | UCL | 7 |  | Catharina Hosp | 3 |
|  | Case Western Reserve Univ | 4 |  | Univ Plymouth | 2 |
| #3 virtual integration environment | Johns Hopkins Univ | 7 | #10 double-blind randomized | ICREA | 6 |
|  | Ist Auxol Italiano | 66 |  | Univ Texas Austin | 3 |
|  | Univ Calif San Diego | 5 |  | Thomas Jefferson Univ | 2 |
|  | Martini Hosp | 4 |  | Childrens Hosp Philadelphia | 2 |
|  | Natl Cheng Kung Univ | 2 |  | Univ Penn | 2 |
| #4 italian consensus conference | Virtual Real Med Inst | 9 | #12 analgesic requirement | McGill Univ | 7 |
|  | Harvard Univ | 7 |  | Cornell Univ | 4 |
|  | Univ S Australia | 6 |  | Baylor Univ | 3 |
|  | Catholic Univ Milan | 5 |  | LaSalle Hosp | 1 |
|  | Univ Verona | 3 |  | Concordia Univ | 1 |
| #5 feasibility study | Univ So Calif | 9 | #16 outpatient pain clinic | Cedars Sinai Med Ctr | 5 |
|  | Univ N Carolina | 7 |  | Univ Hong Kong | 4 |
|  | Emory Univ | 6 |  | Univ Calif Los Angeles | 3 |
|  | Virtually Better Inc | 4 |  | Seoul Natl Univ | 2 |
|  | Univ Florida | 4 |  | Seoul Natl Univ Hosp | 2 |

Table S4. The top ten co-occurrence keywords and the centrality.

| Ranking | Frequency | Keyword | Centrality |
| --- | --- | --- | --- |
| 1 | 670 | virtual reality | 0.09 |
| 2 | 348 | pain | 0.07 |
| 3 | 223 | distraction | 0.07 |
| 4 | 196 | anxiety | 0.06 |
| 5 | 160 | children | 0.05 |
| 6 | 123 | rehabilitation | 0.05 |
| 7 | 121 | management | 0.04 |
| 8 | 119 | therapy | 0.04 |
| 9 | 96 | analgesia | 0.08 |
| 10 | 68 | phantom limb pain | 0.04 |

Table S5. The top ten keywords in each keyword cluster.

| Cluster label | Keyword | Occurrence counts | Cluster label | Keyword | Occurrence counts |
| --- | --- | --- | --- | --- | --- |
| #0 virtual reality intervention | virtual reality | 670 | #5 physical disorder | scale | 47 |
|  | distraction | 223 |  | fear | 38 |
|  | anxiety | 196 |  | exposure therapy | 35 |
|  | children | 160 |  | environment | 33 |
|  | rehabilitation | 123 |  | posttraumatic stress disorder | 13 |
|  | management | 121 |  | memory | 11 |
|  | therapy | 119 |  | graded exposure | 6 |
|  | analgesia | 96 |  | tactile augmentation | 4 |
|  | physical therapy | 67 |  | epidemiology | 3 |
|  | intervention | 66 |  | cope | 3 |
| #1 cortical reorganization | phantom limb pain | 68 | #6 joint excursion | fatigue | 11 |
|  | augmented reality | 43 |  | recovery | 10 |
|  | preception | 37 |  | kinematics | 9 |
|  | brain | 35 |  | chronic back pain | 5 |
|  | reality | 34 |  | arm | 4 |
|  | movement | 31 |  | trunk | 3 |
|  | mirror therapy | 26 |  | heart rate | 3 |
|  | cortical reorganization | 26 |  | avoidance | 2 |
|  | hard | 21 |  | distance perception | 2 |
|  | immersive virtual reality | 20 |  | healthy | 2 |
| #2 neck pain | stroke | 47 | #7 visual distraction decrease | pain | 348 |
|  | technology | 45 |  | cancer pain | 5 |
|  | reliability | 40 |  | belief | 2 |
|  | system | 38 |  | item measure | 2 |
|  | neuropathic pain | 37 |  | ambulation | 1 |
|  | balance | 33 |  | absorption | 1 |
|  | motion | 21 |  | alpha rhythm | 1 |
|  | performance | 18 |  | burn patient | 1 |
|  | symptom | 18 |  | coherence | 1 |
|  | game | 17 |  | cognitive control | 1 |
| #3 aerobic cycling | chronic pain | 53 | #8 postoperative bedside | patient controlled analgesia | 5 |
|  | exercise | 50 |  | cognitive impairment | 3 |
|  | quality of life | 43 |  | morphine | 2 |
|  | randomed controlled trial | 40 |  | adult patient | 2 |
|  | attention | 37 |  | alternative medicine | 1 |
|  | low back pain | 29 |  | childrens distress | 1 |
|  | clinical trial | 23 |  | massagetherapy | 1 |
|  | health | 23 |  | intrathecal morphine | 1 |
|  | physical activity | 23 |  | integrative medicine | 1 |
|  | depression | 18 |  | iliacacompartment block | 1 |
| #4 serious game | surgery | 34 |  |  |  |
|  | validation | 26 |  |  |  |
|  | trial | 25 |  |  |  |
|  | outcm | 24 |  |  |  |
|  | experience | 21 |  |  |  |
|  | prevalence | 21 |  |  |  |
|  | stress | 20 |  |  |  |
|  | behavior | 19 |  |  |  |
|  | anesthesia | 16 |  |  |  |
|  | exposure | 16 |  |  |  |
